# Evaluation of a patient decision aid for initiating disease modifying anti-rheumatic drugs

**DOI:** 10.1186/s13075-016-1138-3

**Published:** 2016-10-28

**Authors:** Ingrid Nota, Constance H. C. Drossaert, Erik Taal, Harald E. Vonkeman, Cees J. Haagsma, Mart A. F. J. van de Laar

**Affiliations:** 1 Department of Psychology, Health and Technology, University of Twente, PO Box 217, 7500 AE Enschede, The Netherlands; 2 Department of Rheumatology and Clinical Immunology, Medisch Spectrum Twente, PO Box 50 000, 7500 KA Enschede, The Netherlands; 3 Department of Rheumatology, Ziekenhuisgroep Twente, PO Box 7600, 7600 SZ Almelo, The Netherlands

**Keywords:** Shared decision-making, Patient participation, Inflammatory arthritis, DMARDs, Patient decision aid

## Abstract

**Background:**

According to international guidelines, treatment of inflammatory arthritis should be based on a shared decision between patient and rheumatologist. Furthermore, patients with inflammatory arthritis have high need of information and want to be more actively involved in medical decision-making. To facilitate shared decision-making and support patients in choosing between disease modifying anti-rheumatic drugs (DMARDs), a web-based patient decision aid (PtDA) was developed. This study evaluated use, appreciation and effect of this PtDA.

**Methods:**

A post-test only study with a historical comparison group was conducted. In a two-year period, all patients diagnosed with rheumatoid arthritis, psoriatic arthritis or ankylosing spondylitis, who were deciding whether to start a (different) DMARD were invited to participate. In the first year, patients received standard information (comparison group). In the second year, patients were referred to the PtDA (intervention group). In both groups, a questionnaire was sent four weeks after consulting the rheumatologist. Patient characteristics included sociodemographic, health-related and preference-related variables. Process measures were for use and appraisal of the PtDA (intervention group only). The primary outcome measure was patients’ perceived role in medical decision-making. Secondary outcome measures comprised satisfaction with the decision-making process and the decision, beliefs about medication, adherence to medication and trust in the physician.

**Results:**

We received 158/232 questionnaires (68 %) from the comparison group and 123/200 (61 %) from the intervention group. The PtDA was used by 69/123 patients (57 %) in the intervention group. Patients who used the PtDA highly appreciated it and perceived it as easy to use and helpful. Relative to the comparison group, patients in the intervention group perceived a more active role in medical decision-making and decisions were more in line with patients’ personal preferences. Other outcomes showed no significant difference between the two groups.

**Conclusion:**

The web-based PtDA was highly appreciated and perceived as helpful for decision-making. Implementation of the PtDA in rheumatology practice was associated with a significantly larger proportion of patients perceiving an active role in medical decision-making and decisions were more in line with patients’ personal preferences. The PtDA can be a valuable aid in improving patient participation in decision-making about DMARDs.

## Background

In recent years, several studies have shown that patients with inflammatory arthritis have a high need of information and want to be more actively involved in medical decision-making [1–8]. Medical decisions in this population focus primarily on the management of the disease with conventional synthetic disease modifying anti-rheumatic drugs (csDMARDs) and biologic disease modifying anti-rheumatic drugs (bDMARDs). When weighing the options, elements to consider include treatment efficacy, approximate time to benefit, possible side effects, current and future risks, cost-effectiveness, route of administration and impact on daily life. Given the preference-sensitive elements of these treatment options, treatment of inflammatory arthritis should be based on a shared decision between the patient and the rheumatologist [9–12].

While desirable, implementing shared decision-making (SDM) in daily clinical practice is challenging. Patients often find it hard to recognise that a decision needs to be made and find it difficult to actively participate in the process to come to an informed values-based decision [8, 13]. Physicians, on the other hand, may be uncomfortable with patient involvement due to a lack of time, self-efficacy or skills [14].

To facilitate SDM and to support patients in making treatment decisions, patient decision aids (PtDAs) have been developed for a wide variety of conditions and treatments [15]. PtDAs make the decision being considered explicit, describe all available treatment options and their pros and cons, and help patients to consider the options from a personal perspective [16, 17].

PtDAs have repeatedly been shown to have a positive impact on patients’ knowledge about options, accurate risk perceptions and feelings of being informed [15]. Moreover, PtDAs have improved patients’ involvement in medical decision-making and have led to decisions that are more in line with patients’ personal preferences [15]. Furthermore, PtDAs sometimes have a positive impact on patients’ satisfaction with decision-making, anxiety, adherence or health outcomes [15]. Although these effects are not likely to be very different, in rheumatology, only a few studies on PtDAs have been reported and their effects have not yet been thoroughly determined [18–20].

With the objective of supporting SDM in rheumatology, we developed a web-based PtDA for initiating csDMARDs and bDMARDs. We have previously described the systematic development of this PtDA [21] using the development process model of the International Patient Decision Aids Standards (IPDAS) [22] combined with user-centred design methods [23, 24].

We conducted a study to evaluate the use, appreciation and effect of the PtDA. The study focused on answering the following research questions: (1) how many patients use the PtDA; (2) what are determinants of use; (3) how do patients appreciate the PtDA; and (4) in comparison to usual care, what is the effect of the PtDA on patients’ perceived role in medical decision-making, satisfaction with the decision and decision-making process, beliefs about medication, adherence and trust in the physician? The primary outcome of the study was the impact of the PtDA on patients’ perceived role in medical decision-making, in comparison to usual care. We also examined use of the PtDA by patients, determinants of use and patients’ appreciation of the PtDA. Furthermore, we explored the impact on satisfaction with the decision and the decision-making process, beliefs about medication, adherence to medication, and trust in the physician.

## Methods

### Description of the PtDA and its integration in clinical practice

The PtDA is intended for patients diagnosed with rheumatoid arthritis (RA), ankylosing spondylitis (AS) or psoriatic arthritis (PsA), who face the decision whether to initiate a DMARD or change to a different DMARD. Based on previous work [25, 26], the tool was designed to enable patients to compare multiple DMARDs with regard to both clinical and practical information with possible consequences for daily life. Furthermore, it aims to support patients in determining treatment preferences, worries and questions and to help patients to express these feelings and questions to the health professionals.

**Fig. 1:**
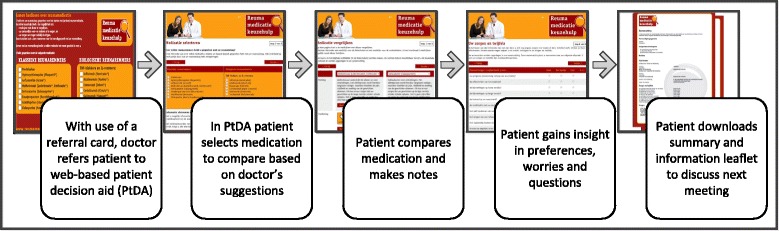
Process of the patient decision aid (PtDA) [21]

Ideally, the PtDA is integrated into the patient pathway, as illustrated in Fig. 1. First, the rheumatologist and patient have an initial conversation about starting a (different) DMARD. During this conversation the rheumatologist refers the patient to the web-based PtDA with use of a card. On this referral card the rheumatologist ticks the DMARDs appropriate for this individual patient. The referral card also states the Internet address of the PtDA. After the conversation, the patient can use the PtDA at home. The web-based PtDA consists of various parts: (1) general information about shared decision-making, inflammatory arthritis (RA, AS and PsA) and DMARDs; (2) an application to compare the particular DMARDs ticked on the card by the rheumatologist; (3) exercises to gain insight into preferences, worries and questions; and (4) a printed summary with the patients’ notes, preferences, worries and questions to be discussed with the rheumatologist at the next consultation. The PtDA is in Dutch, but can be visited via www.reumamedicatiekeuzehulp.nl.

### Study design

A post-test only study with non-equivalent groups was conducted. In this design two nonrandomized groups are compared in a post-test design. In this study, a historical comparison group was used, i.e. the comparison group preceded the intervention group in time.

The study covered a two-year period. In the first year, patients received standard information (comparison group). This standard information consisted of a one page information leaflet briefly describing the DMARD under consideration. It described the intended effect, possible interactions with other medicines, the manner of administration, the follow-up process, a short list with common and important side effects, and possible impact on fertility, pregnancy and breastfeeding. In the second year, patients were referred to the PtDA (intervention group), as described earlier. A questionnaire was sent four weeks after inclusion.

### Patients and procedure

Patients were recruited from two large teaching hospitals in the Netherlands: Medisch Spectrum Twente and Ziekenhuisgroep Twente. Both participating hospitals work according to shared standard operating procedures on how to provide treatment information, which are in line with national guidelines. This practice is uniform across the six rheumatologists in each hospital.

All consecutive patients diagnosed with RA, PsA or AS who visited one of the clinics and discussed initiating a (different) DMARD were informed about the study by their rheumatologist (the same rheumatologists in both years of the study) and asked to give permission for the researcher to contact them. Patients who participated in the comparison group were excluded from participation in the intervention group by the researcher (IN).

Patients who agreed to participate (232 patients in the first year; 200 patients in the second year) were sent the questionnaire by mail, four weeks after the consultation. The questionnaire was accompanied by a letter from their rheumatologist and an informed consent form. The letter stated the decision and the treatment options as discussed at the time of inclusion. Patients were asked to return the completed questionnaires and informed consent form to the university using a prepaid return envelope. After three weeks a reminder was sent to those who had not yet returned the questionnaire.

### Measurements

The questionnaire contained questions on patient characteristics, process measures and outcome measures. Our calculated measurements and the statistics for internal consistency are subsequently described for each measure. Standardized scales were used as much as possible. If there was no Dutch scale available, scales were translated using the forward-backward procedure [27].

#### Patient characteristics

Patient characteristics included sociodemographic, health-related and preference-related variables and treatment options as suggested by the rheumatologist. Sociodemographics included gender, age, marital status, education level and work status.

Health-related variables included diagnosis, time since diagnosis, pain and physical function. Pain was assessed as arthritis-related pain in the prior week with a 0 to 10 numerical rating scale. To measure physical function we used the 10-item Health Assessment Questionnaire version 2 (HAQ-II) [28]. Mean scores range from 0 (minimal loss of function) to 3 (completely disabled) (Cronbach’s α = 0.92).

Preference-related variables included role preference in decision-making about DMARDs and need for information. Role preference was assessed using the Control Preferences Scale (CPS) [29] adapted by Garfield et al. [5]. Patients were asked: “If you are informed about the benefits and risks, who should finally decide about initiating DMARDs?” and could respond on a 5-point scale: 1 (the rheumatologist), 2 (mostly the rheumatologist), 3 (the rheumatologist and me together), 4 (mostly me), and 5 (me alone). The answers were summarized into the values 1 ((mostly) doctor), 2 (shared) and 3 ((mostly) patient), as validated by Degner et al. [29]. Need for information was measured with a 4-item subscale for “need for clarification of medical facts” from the Cologne Patient Questionnaire (CPQ) [30, 31]. Mean scores range from 1 to 5 with a higher score indicating higher need for information (Cronbach’s α = 0.83).

#### Process measures

Process measures evaluated the use and appraisal of the PtDA (intervention group only). Use of the PtDA was assessed by asking respondents if they had (1) received the referral card, (2) received an explanation about the PtDA and (3) had visited the PtDA website. Reasons for not visiting the PtDA website were also assessed. Users of the PtDA were asked which tasks they performed on the PtDA website. Response options are specified in Table 2.

Appraisal of the PtDA was assessed with constructs including subjective impact of the PtDA (five items; Cronbach’s α = 0.84), perceived usefulness (eight items; Cronbach’s α = 0.88), ease of use (four items; Cronbach’s α = 0.87), attractiveness (two items; Cronbach’s α = 0.97), and attitude towards future use (two items; Cronbach’s α = 0.92). The latter four constructs are based on the Technology Acceptance Model (TAM) [32, 33]. Statements related to general and specific elements of the PtDA. Items and response options are specified in Table 4. Mean construct scores range from 1 to 5 with higher scores reflecting higher appraisal. Finally, respondents were asked to rate the overall quality of the PtDA in a range from 0 to 10.

#### Outcome measures

Our primary outcome measure was patients’ perceived role in medical decision-making. Secondary outcome measures comprised satisfaction with the decision and decision-making process. Other secondary outcome measures comprised beliefs about medication, adherence and trust in the physician. All outcome measures specifically focused on the decision that patients discussed with their rheumatologist at the time of inclusion. Rheumatologists registered which treatment options they suggested.

Perceived role in medical decision-making was assessed with the CPS [5, 29]. This measure was also used to assess patients’ preferred role. To assess perceived role, patients were asked: “In your opinion, who finally made this decision?” Patients could respond on a 5-point scale (see previous text). Scores were summarized into the values 1 ((mostly) doctor), 2 (shared) and 3 ((mostly) patient).

Satisfaction with the decision and decision-making process was assessed with 6 scales: satisfaction with participation, satisfaction with amount of received information, informed choice, decision control, satisfaction-uncertainty and consistency with personal values. The scale for satisfaction with participation was developed for this study by the researchers and also consists of five items: “My rheumatologist asked me my opinion on this decision”; “I expressed my opinion on this decision”; “There was enough time for questions”; “I was able to express my questions, worries and doubts” and “My questions were answered”. Mean scale scores range from 1 to 5 with a higher score indicating higher levels of satisfaction with participation (Cronbach’s α = 0.71).

Satisfaction with the amount of information received was assessed with items based on the Satisfaction with Information about Medicines Scale (SIMS) [34]. Respondents rated the amount of information received. It originally includes seventeen items (i.e. information topics), we added four items based on the Dutch legal standards for informed consent: the dosage, frequency of administration, storage and storage life. Response options are: 0 (no, far too little), 1 (no, little too little) and 2 (yes, sufficient), which were recoded into 0 (no) and 1 (yes). The sum scores range from 0 to 21 with higher scores indicating a higher degree of overall satisfaction with the amount of information received (Cronbach’s α = 0.91).

The Dutch Decision Evaluation Scales (DES) [35] were used to assess (1) informed choice: the patient’s perception of the quality of the received information (5 items α = 0.86); (2) decision control: the patient’s perceived level of control over the decision in terms of feelings of regret, anxiety and deciding under pressure (5 items, α = 0.62); and (3) satisfaction-uncertainty: the extent to which a patient is satisfied or still has doubts about the decision (5 items, α = 0.56). Scale scores range from 1 to 5 with higher scores indicating higher levels of informed choice, decision control and higher satisfaction (less uncertainty).

The consistency with personal values scale is a two-item subscale of the Satisfaction With Decision (SWD) scale [36] and measures whether the decision meets personal preferences (Cronbach’s α = 0.79). Mean scale scores for both scales range from 1 to 5 with a higher score indicating higher consistency.

Other secondary outcome measures comprised patients’ beliefs about DMARDs, adherence and trust in the physician. Patients’ beliefs about DMARDs were assessed with the Beliefs about Medicines Questionnaire (BMQ) [37]. The BMQ includes two 5-item subscales assessing patients’ beliefs about the necessity of medication and their concerns about it. Sum scores for both scales range from 5 to 25 with higher scores indicating stronger beliefs (Cronbach’s α = 0.83 and α = 0.73, respectively).

Adherence was measured in participants who self-administered their DMARDs. Participants who had help from a caregiver or who went to the clinic for administration (e.g. intravenous therapy) were excluded from the analysis. We used the 8-item Morisky Medication Adherence (MMA) scale [38]. Sum scores range from 0 to 8 with higher scores representing more adherent behaviour (Cronbach’s α = 0.77).

Trust in the physician was assessed with a 3-item subscale of the Cologne Patient Questionnaire (CPQ) [30, 31]. Mean scale scores range from 1 to 5 with a higher score indicating greater trust (Cronbach’s α = 0.90).

Treatment options suggested by the rheumatologist were registered at the time of inclusion by the rheumatologist after obtaining the patient’s consent. We counted the number of suggested options and grouped it into “one option” or “more than one option”. If combination therapy was suggested (e.g. methotrexate combined with hydroxychloroquine or methotrexate combined with adalimumab) and no alternative options were presented, it was coded as one option.

### Pilot test

Prior to inclusion, we performed a pilot test among patients (*n* = 10) to assess the readability of the questionnaire and acceptability of the time it takes to complete the questionnaire. The test showed that the questionnaire took about 30 minutes to complete, which was acceptable according to the participants. Minor textual adjustments were made following the results of the pilot test.

### Statistical analysis

The Statistical Package for the Social Sciences (version 21.0 IBM SPSS Inc, Chicago, IL, USA) [39] was used to perform all analyses. The Pearson chi-square test (for categorical variables) and *t* test (for continuous variables) were performed to compare characteristics of the comparison group and the intervention group, to examine which factors were associated with use of the PtDA and to evaluate the impact of the PtDA.

## Results

### Patient characteristics

The patients in the comparison group returned 158/232 questionnaires (response rate 68 %), from the intervention group we received 123/200 (response rate 61 %). Within both the comparison group and the intervention group there were no significant differences between respondents and non-respondents in age, gender, diagnosis and amount of options suggested by the rheumatologist.

The comparison group and intervention group did not differ with regard to sociodemographic, health-related or preference-related variables, except for marital status (Table 1). In both groups mean age was about 55 years, most respondents were women and most were diagnosed with RA. Both groups reported having a high need for medical information and most participants preferred shared decision-making.

**Table 1:** Patient-related characteristics (*n* = 281)

Variables	Categories/range	Comparison group (*n* = 158)	Intervention group (*n* = 123)	*P* ^a^
Sociodemographic variables				
Age, years		54 ± 15 (158)	55 ± 13 (123)	n.s.^b^
Gender, % (*n*)	Women	65 % (102)	61 % (75)	
	Men	35 % (56)	39 % (48)	n.s.
Marital status, % (*n*)	Married/living with partner	78 % (121)	89 % (109)	
	Not married/living alone	22 % (34)	11 % (14)	0.02
Education, % (*n*)	Low	26 % (41)	30 % (37)	
	Medium	52 % (81)	50 % (61)	
	High	22 % (34)	20 % (25)	n.s.
Work status, % (*n*)	Employed/studying	67 % (82)	67 % (58)	
	Not employed/not studying	33 % (41)	33 % (29)	n.s.
Health-related variables				
Diagnosis, % (*n*)	Rheumatoid arthritis	76 % (108)	81 % (91)	
	Psoriatic arthritis	19 % (27)	13 % (15)	
	Ankylosing spondylitis	5 % (7)	6 % (7)	n.s.
Years since diagnosis, % (*n*)	<1	37 % (58)	25 % (31)	
	1–5	34 % (52)	41 % (50)	
	6–10	10 % (16)	12 % (14)	
	>10	19 % (29)	22 % (27)	n.s.
Pain (NRS)	Range 0–10	4.7 ± 2,4 (158)	4.7 ± 2,5 (123)	n.s.^b^
Physical function (HAQ-II)	range 0–3	2.17 ± 0.57 (155)	2.13 ± 0.57 (120)	n.s.^b^
Preference-related variables				
Preferred role in decision-making (CPS), % (*n*)	(Mostly) doctor	15 % (23)	8 % (10)	
	Shared	76 % (119)	77 % (94)	
	(Mostly) patient	9 % (14)	15 % (18)	n.s.
Need for information (CPQ)	range 1–5	4.4 ± 0.8 (154)	4.5 ± 0.7 (121)	n.s.^b^

Values are the mean ± standard deviation (number) unless otherwise indicated. Percentages do not include missing cases. ^a^Tested using the Pearson chi-square test unless otherwise indicated. ^b^Tested using the *t* test. *n.s.* not significant (*p* > 0.05), *NRS* numerical rating scale, *HAQ-II* Health Assessment Questionnaire, version 2 [28], *CPS* Control Preference Scale [5, 29], *CPQ* Cologne Preference Questionnaire [30, 31]

### Use of the PtDA

The PtDA was used by 69/123 of respondents (57 %) in the intervention group (Table 2). Many of the non-users (23/53 (43 %) of non-users, which is 19 % of all respondents in the intervention group) mentioned that they had not received a referral card or could not remember having received one. Other reasons for not visiting the PtDA website were not having an Internet connection, having troubles finding the website, no interest and lack of time.

**Table 2:** Use of the patient decision aid (*n* = 123)

Tasks	Percentage (*n*)
Visited the PtDA website	57 % (69)
Reasons for not visiting the PtDA website^a^	
Did not receive referral to the PtDA website or cannot remember	19 % (23)
No Internet	7 % (8)
Could not find PtDA website	6 % (7)
Website did not work	1 % (1)
Not interested	6 % (7)
No time	6 % (7)
Missing	(1)
Received explanation about PtDA	
Yes	69 % (85)
No	22 % (27)
Cannot remember	9 % (11)
Tasks performed on the PtDA website (visitors only; *n* = 69)^b^	
Read general information	94 % (65)
Compared two or more DMARDs	90 % (61)
Made notes in the digital notebook	16 % (11)
Performed exercises about preferences, worries and questions	38 % (26)
Saved or printed an information leaflet	47 % (31)
Read the summary	75 % (50)
Showed the summary to others	38 % (24)
Saved or printed the summary	52 % (34)
Took the summary to their next appointment with the rheumatologist	28 % (18)

Percentages do not include missing cases. ^a^For reasons for not visiting the website, percentages are taken from the total population in the intervention group (*n* = 123). ^b^For tasks performed, percentages are taken from visitors only (*n* = 69). *PtDA* patient decision aid, *DMARD* disease modifying anti-rheumatic drug

Of the PtDA website visitors, 65/69 (94 %) read the general information and 61/69 (90 %) compared two or more DMARDs. The exercises to gain insight into preferences, doubts and questions were performed by 26/69 (38 %) of the users. Furthermore, 31/69 (47 %) of the users saved or printed an information leaflet. The summary with user’s notes, preferences, doubts and questions was read by 50/69 (75 %), shown to others by 24/69 (38 %), saved or printed by 34/69 (52 %) and taken to their next appointment with the rheumatologist by 18/69 (28 %) of the users.

### Determinants of use of the PtDA by patients

When exploring determinants of use of the PtDA, a few significant differences were found between users and non-users (Table 3). Users were significantly younger and higher educated. There were no associations between PtDA use and gender, marital status and employment. Nor was use associated with any of the health-related or preference-related factors. The number of options suggested by the rheumatologist was significantly associated with use of the PtDA; patients who were offered more than one treatment option, were more likely to use the PtDA than those who were offered only one.

**Table 3:** Determinants of use of the patient decision aid (*n* = 123)

Variables	Categories/range	Users PtDA (*n* = 69)	Non-users PtDA (*n* = 53)	*P* ^a^
Sociodemographic variables				
Age, years		52 ± 13 (69)	58 ± 12 (53)	0.003^b^
Gender, % (*n*)	Women	57 % (39)	68 % (36)	
	Men	43 % (30)	32 % (17)	n.s.
Marital status, % (*n*)	Married/living with partner	87 % (60)	91 % (48)	
	Not married/living alone	13 % (9)	9 % (5)	n.s.
Education, % (*n*)	Low	20 % (14)	43 % (23)	
	Medium	51 % (35)	47 % (25)	
	High	29 % (20)	9 % (5)	0.004
Employment, % (*n*)	Employed/studying	65 % (33)	69 % (24)	
	Not employed/studying	35 % (18)	31 % (11)	n.s.
Health-related variables				
Diagnosis, % (*n*)	Rheumatoid Arthritis	81 % (52)	80 % (39)	
	Psoriatic Arthritis	13 % (8)	14 % (7)	
	Ankylosing Spondylitis	6 % (4)	6 % (3)	n.s.
Years since diagnosis, % (*n*)	<1	19 % (13)	32 % (17)	
	1–5	43 % (29)	40 % (21)	
	6–10	13 % (9)	9 % (5)	
	>10	25 % (17)	19 % (10)	n.s.
Pain (NRS)	Range 0–10	4.7 ± 2.5 (69)	4.8 ± 2.8 (53)	n.s.^b^
Physical function (HAQ-II)	range 0–3	2.14 ± 0.70 (69)	2.10 ± 0.71 (53)	n.s.^b^
Preference-related variables				
Preferred role in decision-making (CPS), % (*n*)	(Mostly) doctor	9 % (6)	8 % (4)	
	Shared	78 % (54)	75 % (39)	
	(Mostly) patient	13 % (9)	17 % (9)	n.s.
Need for information (CPQ)	range 1–5	4.6 ± 0.6 (69)	4.5 ± 0.7 (121)	n.s.^b^
Number of treatment options				
Suggested by rheumatologist, % (*n*)	1 option	38 % (23)	73 % (36)	
	>1 option	62 % (38)	27 % (13)	0.000

Values are the mean ± standard deviation (number) unless otherwise indicated. Percentages do not include missing cases. ^a^Tested using the Pearson chi-square tests unless otherwise indicated. ^b^Tested using the *t* test. *PtDA* patient decision aid, *n.s.* not significant (*p* > 0.05), *NRS* numerical rating scale, *HAQ-II* Health Assessment Questionnaire, version 2 [28], *CPS* Control Preference Scale [5, 29], *CPQ* Cologne Preference Questionnaire [30, 31]]

### Appraisal of the PtDA

Overall, users were very positive about the PtDA website (Table 4). Many respondents indicated that they learned a lot from it. They also indicated that it contained new information, helped them to gain insight into preferences, worries and questions, helped them to discuss things with their rheumatologist and helped with making a decision about the medication. They perceived it to be very useful, easy to use, easy to understand and attractive. The general information, the specific pharmaceutical information and the comparison of DMARDs were perceived as most useful. Furthermore, most participants intended to use the PtDA again in the future and would recommend the PtDA to others. The overall quality of the PtDA website received a grade 7.7 on a scale from 0 to 10.

**Table 4:** Appraisal of the patient decision aid (*n* = 69)

	Mostly disagree (1 point)	Neutral (3 points)	Mostly agree (5 points)	Mean ± SD
Subjective impact of the PtDA				
I learned a lot from the PtDA	11 %	17 %	71 %	
The PtDA contained new information	7 %	23 %	70 %	
The PtDA helped me to gain insight into my preferences, worries and questions	9 %	22 %	70 %	
The PtDA helped me in making a decision about medication	11 %	18 %	70 %	
The PtDA helped me in discussing my preferences, worries and questions with my rheumatologist	11 %	28 %	60 %	
Total impact				3.8 ± 0.9
Perceived usefulness				
The PtDA in general was useful	7 %	5 %	88 %	
Reading the general information was useful	2 %	5 %	94 %	
The specific information about DMARDs was useful	4 %	3 %	94 %	
Comparing DMARDs was useful	4 %	8 %	89 %	
Making notes in the digital notebook was useful	22 %	51 %	28 %	
The exercises about preferences and worries were useful	11 %	29 %	59 %	
The list of frequently asked questions was useful	12 %	32 %	56 %	
Total usefulness				4.0 ± 0.7
Perceived ease of use				
The website is easy to use	5 %	3 %	92 %	
The information is easy to understand	2 %	6 %	92 %	
The time the PtDA takes to finish is acceptable	5 %	5 %	90 %	
The structure of the website is logical	10 %	11 %	79 %	
Total ease of use				4.5 ± 0.8
Attractiveness				
The colour of the website is pleasant	2 %	17 %	81 %	
The font on the website is pleasant	4 %	9 %	87 %	
Total attractiveness				4.4 ± 0.9
Attitude towards future use				
I would use the PtDA again in the future	5 %	3 %	91 %	
I would recommend the PtDA to others	2 %	7 %	89 %	
Total attitude				4.6 ± 0.9
Overall grade regarding the quality of the PtDA (range 0–10)				7.7 ± 0.9

Percentages do not include missing cases. *PtDA* patient decision aid, *DMARD* disease modifying anti-rheumatic drug

### Impact of the PtDA

Relative to the comparison group, patients in the intervention group perceived significantly less often that the doctor decided about initiating DMARDs, and more often that they made the final decision about initiating DMARDs (Table 5). With regard to the secondary outcome measures, we found that patients in the intervention group regarded the decision to be significantly more consistent with their personal preferences than patients in the comparison group. Finally, more participants in the intervention group were offered more than one medication option compared to patients in the comparison group, 46 % vs 12 %; *p* < 0.05, respectively. For all other variables no significant differences were found between the groups.

**Table 5:** Impact of the patient decision aid (*n* = 281)

	Comparison group* *n* = 158	Intervention group* *n* = 123	*P* ^a^
Perceived role in decision-making (CPS), % (*n*)			
(Mostly) doctor	25 % (39)	14 % (17)	
Shared	70 % (111)	73 % (90)	
(Mostly) patient	5 % (8)	13 % (16)	0.01^b^
Satisfaction with decision and decision-making process			
Satisfaction with participation (range 1–5)	4.6 ± 0.6 (144)	4.6 ± 0.6 (115)	n.s.
Satisfaction with received information (range 0–21)	15.7 ± 4.9 (130)	15.3 ± 5.7 (106)	n.s.
Informed choice (DES) (range 1–5)	4.2 ± 1.0 (145)	4.3 ± 0.9 (115)	n.s.
Decision-control (DES) (range 1–5)	4.6 ± 0.5 (146)	4.6 ± 0.7 (114)	n.s.
Satisfaction-uncertainty (DES) (range 1–5)^c^	4.0 ± 0.8 (147)	4.1 ± 0.7 (116)	n.s.
Consistency with personal values (SWD)	4.2 ± 1.0 (148)	4.5 ± 0.8 (112)	0.02
Other categories			
Beliefs about medication - necessity (range 5–25)	18.6 ± 4.5 (137)	19.6 ± 4.6 (87)	n.s.
Beliefs about medication - concerns (range 5 − 25)	13.8 ± 4.1 (136)	12.9 ± 4.9 (90)	n.s.
Medication adherence (MMAS) (range 0 − 8)^d^	7.2 ± 1.4 (129)	7.2 ± 1.4 (102)	n.s.
Trust in physician (CPQ) (range 1 − 5)	4.8 ± 0.5 (155)	4.8 ± 0.4 (120)	n.s.
Number of treatment options suggested by rheumatologist, % (*n*)			
1 option	88 % (137)	54 % (60)	
> 1 option	12 % (18)	46 % (51)	0.000

*Values are the mean ± standard deviation (number) unless otherwise indicated. ^a^Tested using the *t* test unless otherwise indicated. ^b^Tested using the Pearson chi-square test. ^c^Higher scores indicate less uncertainty and higher satisfaction. ^d^Adherence not assessed in patients that get professional assistance with administrating injections or go to the hospital for intravenous medication

*n.s.* not significant (*p* > 0.05), *CPS* Control Preference Scale [5, 29], *DES* Decision Evaluation Scales [35], *SWD* Satisfaction With Decision scale [36], *BMQ* Beliefs about Medication Questionnaire [37], *MMAS* Morisky Medication Adherence Scale [38], *CPQ* Cologne Preference Questionnaire [30, 31]

## Discussion

This study was conducted to evaluate the use, appraisal and impact of a PtDA for initiating DMARDs in patients with rheumatic diseases. The PtDA is designed to improve patient participation by supporting patients in determining treatment preferences, worries and questions and by endorsing them to express these feelings and questions to their health professionals. The study demonstrated that patients perceived the PtDA as very helpful in the decision-making process. Our primary research question focused on the impact of the PtDA on perceived role in medical decision-making. Relative to the comparison group, patients in the intervention group perceived a more active role in medical decision-making. Furthermore, decisions were more in line with patients’ personal preferences. We found no differences between groups in satisfaction with the decision-making process, beliefs about medication, adherence or trust in the physician. However, this may be due to ceiling effects or limited psychometric quality of some of the instruments used [40]. Generally, our results are in line with the impact of other PtDAs, as was shown in a recent systematic review [15].

By developing the PtDA in co-creation with patients and health professionals we aimed to develop a user-friendly PtDA that closely fits the needs of all users and consequently eased adoption and implementation. This study demonstrated that patients appreciated the PtDA highly and perceived it as useful, usable and helpful in the decision-making process.

The PtDA was used by 57 % of the patients in the intervention group who had returned the questionnaire. Users were mostly younger and higher educated patients. Compared to other studies on PtDAs in routine practice and in clinical trials, our patient user rates are high. In other routine practice studies, patients’ use of PtDAs varied between 25 % and 37 % [41–43]. Clinical trials report much higher patient user rates, varying from 49 % to 85 % [44–46].

A recent systematic review suggests that adoption and implementation of PtDAs using a referral model (i.e. health professionals inviting eligible patients to use the PtDA) is often challenged by indifference on the part of health professionals [14]. This indifference may stem from a lack of confidence in the content of PtDAs and concerns about disruption of established workflow [14]. However, we believe that the relatively high percentage of patients who used the PtDA in our study may partly be explained by the active referral by the rheumatologists. Although we did not specifically assess factors enhancing system adoption in this study, we believe that the high referral and usage rates could be attributed to the iterative and extensive involvement of patients and health professionals during the development process [47]. To determine how to further increase the referral rates to the PtDA, we recently conducted a focus group study with health professionals, the results of which are currently being analysed. Likewise, to further increase patients’ PtDA use it should be investigated how the tool can be further adapted to the needs of older and lower educated patients.

Notably, we observed a significant difference between the comparison group and the intervention group in the number of treatment options offered by the rheumatologist. This might have biased the findings because when patients are offered more than one option, or when options are more explicitly discussed, patients might (automatically) feel that their role in medical decision-making is larger. However, the question remains why the amount of options offered and registered by the rheumatologists was higher in the second period of the study (intervention group). There have been no apparent changes in the availability of DMARDs, and the way rheumatologists were asked to register the options offered was identical in both periods. Interviewing some of the participating rheumatologists revealed that the referral card and PtDA may have prompted the rheumatologists to more explicitly discuss options with patients and consequently more accurately register the options offered for the study.

Unfortunately, we cannot compare the effect on number of options offered to results for other PtDAs, because this is not a common measure in PtDA evaluations. Previously studied PtDAs have largely focused on decisions on whether or not to initiate a treatment or on choosing between a predefined limited number of treatment options. Widely studied examples include decisions like “Should I have chemotherapy for early-stage breast cancer?” and “Should I have breast-conserving surgery or a mastectomy for early-stage breast cancer?” In rheumatology, previous PtDAs focused on the decision on whether or not to initiate one specific DMARD or a particular class of DMARDs [18, 19]. Compared to these previously studied PtDAs, our PtDA encompasses many different treatment options. To reduce the potentially overwhelming number of choices and to eliminate all inappropriate options, we chose to let the rheumatologist preselect which DMARDs are appropriate choices for the individual patient at that specific moment. To our knowledge, this innovative flexible referral model has not previously been studied.

The main strength of this study is its virtual implementation of a PtDA in daily clinical practice. However, due to limitations inherent in the study design, some caution is needed when interpreting our results. First, the post-test only study with a non-equivalent historical comparison group is susceptible to the internal validity of selection; any prior differences between the groups may have affected the outcome of the study. Yet, despite this limitation we chose this study design deliberately in order to reduce contamination effects. If patients had been randomized to a condition, namely PtDA or standard information, physicians would have been exposed to both conditions simultaneously, which might have influenced their behaviour. Second, although we included many variables in our study, it remains difficult to control for all confounding variables. We realize that in this study we did not evaluate merely the effect of the PtDA. Introducing the PtDA obviously affected the healthcare system and the daily workflow of health professionals. Therefore, some caution is needed with causal interpretations of our results. Finally, due to non-response, our results might have been biased. It is likely that the patients who do not need to participate in medical decision-making or in using a PtDA are less interested in responding to a questionnaire about this subject.

Future multi-centre randomized trials need to be conducted to further study the impact of this PtDA and to compare the impact of this PtDA with other SDM interventions. A longitudinal study is needed to reveal what the impact is on the number of sessions and on cost-effectiveness. Furthermore, more research is needed to determine how to involve lower educated patients and patients in different age groups in medical decision-making.

## Conclusion

This study was conducted to evaluate use, appraisal and impact of a PtDA for making decisions about initiating DMARDs. The PtDA was used by the majority of the respondents, was highly appreciated and was perceived as helpful in the decision-making process. Relative to the comparison group, patients perceived a more active role in medical decision-making and felt the final choice to be more consistent with their personal preferences. From this study we can conclude that this PtDA can be a valuable aid in improving patient participation in medical decision-making about DMARDs.

## Abbreviations

AS: Ankylosing spondylitis
bDMARDs: biologic disease modifying anti-rheumatic drugs
BMQ: Beliefs about Medicines Questionnaire
CPQ: Cologne Patient Questionnaire
CPS: Control Preferences Scale
csDMARDs: conventional synthetic disease modifying anti-rheumatic drugs
DMARDs: Disease modifying anti-rheumatic drugs
HAQ-II: Health Assessment Questionnaire version 2
IPDAS: International Patient Decision Aids Standards
PsA: Psoriatic arthritis
PtDA: Patient decision aid
RA: Rheumatoid arthritis
SDM: Shared decision-making
